# Drug preparation, injection-related infections, and harm reduction practices among a national sample of individuals entering treatment for opioid use disorder

**DOI:** 10.1186/s12954-024-00939-6

**Published:** 2024-01-19

**Authors:** Laura R. Marks, Michael J. Durkin, Kelly Ayres, Matthew Ellis

**Affiliations:** 1 Division of Infectious Disease, Washington University School of Medicine, Campus Box 8051, 4523 Clayton Avenue, St. Louis, MO USA; 2 Department of Psychiatry, Washington University School of Medicine, St. Louis, MO USA

**Keywords:** Substance use disorder, Harm reduction, Patients who inject drugs

## Abstract

**Background:**

The rise in injection drug use in the USA has led to an increase in injection site infections. We performed a national survey of people who use drugs to evaluate common drug use preparation, harm reduction practices, and experiences with injection site infections.

**Methods:**

A survey was disseminated to members of the Survey of Key Informants’ Patients Program from 2021 to 2022 and distributed to patients 18 years or older newly entering one of 68 substance use disorder treatment programs across the USA with a primary diagnosis of an opioid use disorder. Participants were surveyed about practices when preparing and using drugs, along with self-reported infections and drug use complications.

**Results:**

1289 participants responded to the survey. Sexually transmitted infections were common, with 37.6% reporting ever having had any sexually transmitted infection. Injection-associated infections had affected 63.4% of participants who had ever used injection drugs. Many respondents reported not seeking professional medical assistance for infection management, including 29% draining abscesses without seeking medical care and 22.8% obtaining antibiotics through non-healthcare sources. Non-sterile injection practices included sharing needles with others who were febrile or ill (18%), using needles previously used to drain wounds/abscesses (9.9%) for subsequent injection drug use, and licking needles (21.2%).

**Conclusion:**

Patients entering treatment for opioid use disorder reported a high burden of infectious diseases. A number of easily-modifiable high risk behaviors for developing injection-related infections were identified. Efforts are needed to disseminate targeted harm reduction education to PWID on how to reduce their risks for injection-related infections.

## Introduction

The opioid crisis is driving an epidemic of infectious diseases among people who inject drugs (PWID) including outbreaks of human immunodeficiency virus (HIV) [1–3], viral hepatitis [4], and bacterial and fungal infections [5]. The most frequently described infectious complication of injection drug use is skin and soft tissue infections [6, 7], with a lifetime incidence of up to 68%, and life threatening osteoarticular and endovascular infections are increasing in prevalence [8].

A growing body of the literature demonstrates that pathogens can be transmitted through shared injection equipment [9], non-sterile drugs [10–12], solvents and unsanitary injection practices [13, 14]. Whole-genome sequencing studies have demonstrated transmission of invasive *S. aureus* infections within the injection drug use network [15–17]. Furthermore, these risks may be modifiable. Epidemiologic studies reveal that PWID who engage in higher-risk behaviors have an increased risk of skin and soft tissue infections [18, 19]. Harm reduction education targeting safer injection practices may provide a key tool in the prevention of these infections [20, 21].

Clinicians caring for people who use drugs must be aware of current injection drug use practices in order to provide targeted and relevant education on safer injection techniques. We used a nationally recognized Survey of Key Informants' Patients (SKIP) Program administered through the Researched Abuse, Diversion and Addiction-Related Surveillance (RADARS®) System [22, 23] to collect data on current drug use preparation and harm reduction practices among people entering treatment for opioid use disorder (OUD) in 2021.

## Methods

Participation in this study was subordinate to admission into the ongoing, serial cross-sectional Survey of Key Informants' Patients (SKIP) Program which has been described previously [22]. The SKIP Program has served as a unique epidemiological tool amid the opioid epidemic for over a decade, gathering data from participants who are entering treatment centers for opioid use disorder and has been validated through the Researched Abuse, Diversion and Addiction-Related Surveillance (RADARS®) System [22, 23]. Each of these treatment centers is supplied with anonymous paper surveys (i.e., no identifying information) and directed to provide one survey to unique individuals (‘patients’) 18 years or older newly entering the facility with a primary diagnosis of OUD as defined by DSM V criteria. Respondents are given a $20 Wal-Mart gift card for completion of the survey, along with a self-addressed stamped envelope to return the survey once completed. The present analysis includes data from respondents who entered any one of 68 nationally distributed treatment centers from fourth quarter 2021 through fourth quarter 2022.

### Sociodemographic characteristics

Participants reported their gender, age (in years), race (coded as non-Hispanic White, non-Hispanic Black, and other), housing status in the past month (street living, shelter/respite care, staying with family or friends, rent/own), self-identified residential status (urban/rural) and route of drug use (ever injected drugs *n* = 728 vs no history of injection drug use *n* = 561) amounting to a total sample size of *n* = 1289.

Group-wise trends in drug use were calculated and compared to ascertain injection drug use-specific differences. Relevant drug use strata included, healthcare coverage, educational attainment, type of substances used in addition to opioids, other comorbid infectious diseases health conditions (including history of sexually transmitted infections, HIV, and HCV), drug use preparation and harm reduction practices, and injection drug use-associated bacterial and fungal infections.

### Drug use and harm reduction practices

Participants who reported injecting drugs were asked (yes/no) if they had ever engaged in any of the following harm reduction practices: used alcohol pads to clean an injection site, cleaned previously used needles with bleach or alcohol, reused needles, reused needles they previously used to drain an infection to subsequently inject drugs, shared needles with someone else that might have a fever or was sick, used saliva to lubricate a dull needle, used lemon juice or other acidic fruit juice to dissolve drugs, or none of the above.

### Injection drug use-associated bacterial and fungal infections

Participants who reported injecting drugs were then asked (yes/no) which of the following infections they had ever had as a result of injection: skin redness at an injection site (cellulitis), an abscess or ‘boil’ at an injection site (abscess), an open wound at an injection site, a bloodstream infection or sepsis, an infection of the heart valve (endocarditis), or none of the above.

Analysis of the survey was performed using SPSS version 27. Categorical variables were compared using *χ*^2^, and differences were considered significant at *p* < 0.05.

## Results

### Respondent demographics

As shown in Table 1, 56.5% of this sample of individuals entering treatment for OUD reported a lifetime history of injection drug use (IDU). Injection drug use was significantly more prevalent among white individuals, those aged 25–34, sexual minorities, and individuals living in rural areas. Social determinants associated with an increased likelihood of injection included a lack of stable housing, receiving income from friends/family, having no health insurance, and having an educational attainment less than some college. Lifetime history of psychiatric illness as well as a history of suicide attempts was also associated with increased injection behaviors, as well as comorbid stimulant, marijuana, and anxiolytic use.

**Table 1:** Sociodemographics of survey respondents stratified by route of prior drug use

	No injection drug use (*n* = 561)		Ever injected drugs (*n* = 728)		Total (*N* = 1289)		sig. (*χ*^2^)
*Demographics*							
Female	202	36.9%	264	36.8%	466	36.8%	0.953
White	299	56.7%	529	75.8%	828	67.6%	< .001
Black	137	26.0%	62	8.9%	199	16.2%	< .001
Latinx	49	9.3%	57	8.2%	106	8.7%	0.485
Other race	39	7.0%	29	4.0%	68	5.3%	0.018
Sexual minority (i.e., non-heterosexual)	59	10.8%	129	17.9%	188	14.8%	< .001
Transgender or gender non-binary	7	1.3%	16	2.3%	23	1.9%	0.211
*Age at survey completion*							
18–24	57	10.2%	48	6.6%	105	8.1%	0.020
25–34	200	35.7%	318	43.7%	518	40.2%	0.004
35–44	175	31.2%	238	32.7%	413	32.0%	0.568
45+	129	23.0%	124	17.0%	253	19.6%	0.008
*Past month housing status*							
Temporarily with friend/family member	115	29.1%	195	35.2%	310	32.7%	0.049
Safe Haven/shelter	33	8.4%	68	12.3%	101	10.6%	0.054
Rent/own	197	49.9%	114	20.6%	311	32.8%	< .001
Street dwelling	18	4.6%	93	16.8%	111	11.7%	< .001
Other	78	13.9%	205	28.2%	283	22.0%	< .001
*Urbanicity*							
Urban	323	59.2%	353	50.1%	676	54.0%	0.001
Suburban	112	20.5%	145	20.6%	257	20.5%	0.981
Rural	116	21.2%	210	29.8%	326	26.1%	< .001
*Primary source of income*							
Employed/retired	275	50.4%	256	35.7%	531	42.0%	< .001
Public assistance	86	15.8%	101	14.1%	187	14.8%	0.409
Friend/family	93	17.0%	184	25.7%	277	21.9%	< .001
Other	119	21.8%	212	29.6%	331	26.2%	0.002
*Healthcare coverage*							
None	140	25.9%	258	36.3%	398	31.8%	< .001
Covered under another individual	30	5.5%	16	2.3%	46	3.7%	0.002
Medicare/Medicaid	252	46.6%	365	51.4%	617	49.3%	0.091
Private	82	15.2%	27	3.8%	109	8.7%	< .001
VA/military healthcare	7	1.3%	5	0.7%	12	1.0%	0.289
*Educational attainment*							
Less than high school	84	15.0%	143	19.6%	227	17.6%	0.031
High school/GED	248	44.4%	379	52.1%	627	48.7%	0.006
Some college	175	31.3%	167	22.9%	342	26.6%	< .001
Any post-secondary accreditation	81	14.5%	78	10.7%	159	12.4%	0.041
*Comorbid conditions*							
Lifetime history of psychiatric diagnosis/treatment	177	32.8%	395	56.4%	572	46.1%	< .001
Chronic pain (i.e., pain lasting 3 months or longer)	241	43.0%	362	49.7%	603	46.8%	0.644
Lifetime history of suicide attempt	111	20.6%	261	36.6%	372	29.7%	< .001
Lifetime history of an opioid overdose	151	26.9%	461	63.3%	612	47.5%	< .001
Past month non-opioid use*							
Nicotine/tobacco	448	92.8%	643	94.4%	1091	93.7%	0.248
Marijuana	233	41.5%	363	49.9%	596	46.2%	0.003
Alcohol (> 4 times in one day)	146	30.2%	197	28.9%	343	29.5%	0.632
Crystal meth	85	17.6%	335	49.2%	420	36.1%	< .001
Anxiolytics	96	17.1%	180	24.7%	276	21.4%	< .001
Crack/cocaine	92	19.0%	194	28.5%	286	24.6%	< .001
Muscle relaxants	74	13.2%	109	15.0%	183	14.2%	0.363
Antidepressants	53	9.4%	71	9.8%	124	9.6%	0.854
Prescription stimulants	42	7.5%	81	11.1%	123	9.5%	0.027
Prescription sleep medications	48	8.6%	78	10.7%	126	9.8%	0.196
Hallucinogens	34	7.0%	51	7.5%	85	7.3%	0.771
MDMA	25	5.2%	57	8.4%	82	7.0%	0.036
*Sexually transmitted infections*							
Any STI (including HIV/HCV)	130	23.2%	355	48.8%	485	37.6%	< .001
Chlamydia	79	14.10%	161	22.1%	240	18.6%	< .001
Gonorrhea	41	7.30%	112	15.4%	153	11.9%	< .001
Syphilis	19	3.4%	30	4.1%	49	3.8%	0.494
HPV	9	1.6%	32	4.4%	41	3.2%	0.005
Herpes	11	2.0%	40	5.5%	51	4.0%	0.001
Lifetime history of trading sex for drugs	83	15.1%	267	37.3%	350	27.6%	< .001
*HCV status*							
HCV positive	9	2.0%	176	26.1%	185	16.3%	< .001
HCV negative tested within the past three months	138	30.2%	188	27.9%	326	28.8%	0.393
HCV negative tested over three months ago	149	32.6%	175	25.9%	324	28.6%	0.015
Never tested for HCV	161	35.2%	136	20.1%	297	26.2%	< .001
*HIV status*							
HIV positive	7	1.5%	18	2.7%	25	2.2%	0.173
HIV negative tested within the past 3 months	150	31.6%	279	41.3%	429	37.3%	< .001
HIV negative tested over 3 months ago	170	35.8%	279	41.3%	449	39.0%	0.060
Never tested for HIV	148	31.2%	100	14.8%	248	21.5%	< .001

*HCV* hepatitis C Virus, *HIV* human immunodeficiency virus

*Patients may report more than one type of substance use in addition to opioids

### History of HIV, HCV, and other sexually transmitted infections (STI)

Sexually transmitted infections were twice as common among individuals with a history of IDU (48.8% vs. 23.2%, *p* < 0.001). In addition, IDU was also associated with an increased prevalence of trading sex for drugs (37.3% vs. 15.1%, *p* < 0.001). While a substantial fraction of the sample did not recall ever having been tested for HCV (26.2%) or HIV (21.5%), individuals with IDU were more likely to report having received testing.

### Characteristics and harm reduction practices of survey respondents who used injection drugs

Of the 728 individuals who answered yes to having used injection drugs, 63.4% (462/728) reported a prior experience with any type of injection drug use-associated bacterial or fungal infection. Table 2 examines the sociodemographic characteristics associated with the development of these injection drug use-associated bacterial or fungal infections. Street dwelling, being on Medicare or Medicaid, psychiatric illness, and trading sex for drugs were significantly associated with a history of infection. Comorbid use of crystal meth was also associated with development of any type of bacterial or fungal infection (*p* = 0.003). Self-reported injection site infections were more common among people with hepatitis C virus (*p* < 0.001), though not among people living with HIV (*p* = 0.472).

**Table 2:** Sociodemographic characteristics of survey respondents who used injection drugs and associations with development of any injection drug use-associated bacterial or fungal infection

	No infection (*n* = 266)		Any infection (*n* = 462)		Total (*N* = 728)		sig. (*χ*^2^)
*Demographics*							
Female	98	37.3%	166	36.5%	264	36.8%	0.835
White	177	70.0%	352	79.1%	529	75.8%	0.007
Black	32	12.6%	30	6.7%	62	8.9%	0.008
Latinx	25	9.9%	32	7.2%	57	8.2%	0.212
Other race	12	4.6%	17	3.7%	29	4.0%	0.565
Sexual minority (i.e., non-heterosexual)	50	19.0%	79	17.2%	129	17.9%	0.543
Transgender or gender non-binary	3	1.2%	13	2.9%	16	2.3%	0.133
*Age at survey completion*							
18–24	20	7.5%	28	6.1%	48	6.6%	0.445
25–34	107	40.2%	211	45.7%	318	43.7%	0.154
35–44	87	32.7%	151	32.7%	238	32.7%	0.995
45+	52	19.5%	72	15.6%	124	17.0%	0.171
*Past month housing status*							
Temporarily with friend/family member	67	35.8%	128	34.9%	195	35.2%	0.825
Safe Haven/shelter	25	13.4%	43	11.7%	68	12.3%	0.575
Rent/Own	44	23.5%	70	19.1%	114	20.6%	0.220
Street dwelling	21	11.2%	72	19.6%	93	16.8%	0.012
Other	69	25.9%	137	29.7%	206	28.3%	0.895
*Urbanicity*							
Urban	128	49.8%	225	50.2%	353	50.1%	0.915
Suburban	50	19.5%	95	21.2%	145	20.6%	0.580
Rural	80	31.1%	130	29.0%	210	29.8%	0.555
*Primary source of income*							
Employed/retired	100	38.5%	156	34.1%	256	35.7%	0.245
Public assistance	28	10.8%	73	16.0%	101	14.1%	0.054
Friend/family	72	27.7%	112	24.5%	184	25.7%	0.348
Other	68	26.2%	144	31.5%	212	29.6%	0.131
*Healthcare coverage*							
None	103	39.6%	155	34.4%	258	36.3%	0.168
Covered under another individual	7	2.7%	9	2.0%	16	2.3%	0.549
Medicare/Medicaid	120	46.2%	245	54.4%	365	51.4%	0.033
Private	14	5.4%	13	2.9%	27	3.8%	0.094
VA/Military Healthcare	1	0.4%	4	0.9%	5	0.7%	0.439
*Educational attainment*							
Less than high school	44	16.5%	99	21.4%	143	19.6%	0.110
High school/GED	136	51.1%	243	52.6%	379	52.1%	0.702
Some college	64	24.1%	103	22.3%	167	22.9%	0.585
Any post-secondary accreditation	32	12.0%	46	10.0%	78	10.7%	0.384
*Comorbid conditions*							
Lifetime history of psychiatric diagnosis/treatment	127	47.7%	268	58.0%	395	54.3%	0.004
Chronic pain (i.e., pain lasting 3 months or longer)	131	49.2%	231	50.0%	362	49.7%	0.708
Lifetime history of suicide attempt	95	36.7%	166	36;5%	261	36.6%	0.958
Lifetime history of an opioid overdose	154	57.9%	307	66.5%	461	63.3%	0.021
*Past month non-opioid use*							
Nicotine/tobacco	241	94.5%	402	94.4%	643	94.4%	0.937
Marijuana	129	48.5%	234	50.6%	363	49.9%	0.576
Alcohol (> 4 times in one day)	79	31.0%	118	27.7%	197	28.9%	0.361
Crystal meth	107	42.0%	228	53.5%	335	49.2%	0.003
Anxiolytics	66	24.8%	114	24.7%	180	24.7%	0.967
Crack/cocaine	81	31.8%	113	26.5%	194	28.5%	0.143
Muscle relaxants	38	14.3%	71	15.4%	109	15.0%	0.694
Antidepressants	26	9.8%	45	9.7%	71	9.8%	0.988
Prescription stimulants	31	11.7%	50	10.8%	81	11.1%	0.731
Prescription sleep medications	25	9.4%	53	11.5%	78	10.7%	0.384
Hallucinogens	14	5.5%	37	8.7%	51	7.5%	0.125
MDMA	27	8.6%	35	8.2%	57	8.4%	0.851
*Sexually transmitted infections*							
Any STI (including HIV/HCV)	107	40.2%	248	53.7%	355	48.8%	< .001
Chlamydia	51	19.2%	110	23.8%	161	22.1%	0.147
Gonorrhea	33	12.4%	79	17.1%	112	15.4%	0.091
Syphilis	11	4.1%	19	4.1%	30	4.1%	0.988
HPV	5	1.9%	27	5.8%	32	4.4%	0.012
Herpes	9	3.4%	31	6.7%	40	5.5%	0.058
Lifetime history of trading sex for drugs	73	27.4%	194	42.0%	267	36.7%	< .001
*HCV status*							
HCV positive	40	16.6%	136	31.1%	176	26.1%	< .001
HCV negative tested within the past 3 months	74	30.7%	114	26.3%	188	27.9%	0.218
HCV Negative tested over three months ago	69	28.6%	106	24.4%	175	25.9%	0.232
Never tested for HCV	58	24.1%	78	18.0%	136	20.1%	0.059
*HIV status*							
HIV positive	5	2.1%	13	3.0%	18	2.7%	0.472
HIV negative tested within the past three months	103	42.6%	176	40.6%	279	41.3%	0.611
HIV negative tested over three months ago	98	40.5%	181	41.7%	279	41.3%	0.759
Never tested for HIV	36	14.9%	64	14.7%	100	14.8%	0.964
*Injection drug use*							
Prescription opioids	66	24.8%	208	45.0%	274	37.6%	< .001
Heroin/illicit fentanyl	207	77.8%	438	94.8%	645	88.6%	< .001
Amphetamines	25	9.4%	131	28.4%	156	21.4%	< .001
Methamphetamine	151	56.8%	337	72.9%	488	67.0%	< .001
Crack/cocaine	82	30.8%	231	50.0%	313	43.0%	< .001
*Harm reduction practices*							
Any harm reduction practice	172	64.7%	384	83.1%	556	76.4%	< .001
Used alcohol pads to clean an injection site	141	53.0%	299	64.7%	440	60.4%	0.002
Cleaned used needles with bleach or alcohol	111	41.7%	307	66.5%	418	57.4%	< .001
*Non-sterile drug preparation practices*							
Reused needles	144	54.1%	381	82.5%	525	72.1%	< .001
Reused needles you previously used to drain an infection to drain or treat an infection	10	3.8%	62	13.4%	72	9.9%	< .001
Shared needles with someone else who had a fever might have had a fever or was sick	16	6.0%	115	24.9%	131	18.0%	< .001
Shared needles with other individuals	76	28.6%	270	58.4%	346	47.5%	< .001
Used saliva to lubricate a dull needle	26	9.8%	128	27.7%	154	21.2%	< .001
Used lemon or other acidic fruit juice to prepare drugs prepare drugs for injection	24	9.0%	146	31.6%	170	23.4%	< .001

*HCV* hepatitis C Virus, *HIV* human immunodeficiency virus

*Patients may report more than one type of substance use in addition to opioids

The most commonly reported bacterial and fungal infections among respondents who reported injecting drugs were skin and soft tissue infections including cellulitis (358/728, 49.2%) and abscesses (306/728 42.9%), with a minority reporting bloodstream infections (74/728, 10.2%) or a history of infective endocarditis (31/728, 4.3%).

Injection drug use preparation and harm reduction practices varied widely among survey participants. Among those who had ever injected drugs 556 (76.4%) reported having ever used any type of harm reduction technique including cleaning injection sites with alcohol pads prior to drug use or cleaning used needles with bleach or alcohol. Interestingly, respondents that reported have ever engaged in some form of harm reduction practice focused on infection prevention (such as cleaning injection sites) were also more likely to have ever experienced any type of injection-associated infection (cellulitis, abscess, bloodstream infections, and/or endocarditis) (Table 3). This same group also reported an increased rate of having previously engaged in any type of non-sterile drug use practices (Table 3).

**Table 3:** Characteristics of injection site infections and drug preparation practices among survey respondents reported using harm reduction techniques

	No harm reduction practice (*n* = 172)		Any harm reduction practice (*n* = 556)		Total (*N* = 728)		sig. (*χ*^2^)
*Non-sterile drug preparation practices*							
Reused needles	88	51.2%	437	78.6%	525	72.1%	< .001
Reused needles you previously used to drain or treat an infection	9	5.2%	63	11.3%	72	9.9%	0.019
Shared needles with someone else who might have had a fever or was sick	16	9.3%	115	20.7%	131	18.0%	< .001
Shared needles with other individuals	59	34.3%	287	51.6%	346	47.5%	< .001
Used saliva to lubricate a dull needle	20	11.6%	134	24.1%	154	21.2%	< .001
Used lemon or other acidic fruit juice to prepare drugs for injection	19	11.0%	151	27.2%	170	23.4%	< .001
*Infections arising from injection drug use*							
Skin redness (cellulitis) at injection site	51	29.7%	307	55.2%	358	49.2%	< .001
An abscess or boil at injection site	58	33.7%	248	44.6%	306	42.0%	0.012
An open wound at injection site	17	9.9%	99	17.8%	116	15.9%	0.013
A bloodstream infection or sepsis	6	3.5%	68	12.2%	74	10.2%	< .001
Endocarditis (infection of the heart valve)	0	0.0%	31	5.6%	31	4.3%	0.002
None of the above	84	48.8%	155	27.9%	239	32.8%	< .001
*Infection management*							
Drained the infection without seeking medical care	27	15.7%	184	33.1%	211	29.0%	< .001
Received medical care at a hospital or healthcare facility	42	24.4%	214	38.5%	256	35.2%	< .001
Received medical care outside of a hospital or a healthcare facility	13	7.6%	80	14.4%	93	12.8%	0.019
Purchased/received antibiotics through clinic/pharmacy/hospital	17	9.9%	150	27.0%	167	22.9%	< .001
Obtained antibiotics through other sources	24	14.0%	142	25.5%	166	22.8%	0.002

Types of non-sterile drug use practices varied widely, but included dissolving drugs in fruit juice (23.4%), the reuse of needles (72.1%), sharing needles with others who were febrile or ill (18%), reuse of needles previously used to drain wounds/abscesses to subsequently inject drugs (9.9%), and licking needles (21.2%).

Respondents were surveyed about their use of medical services for infection management and where they received care or if they self-treated instead. While some respondents did receive medical care at a hospital or healthcare facility (35.2%) the use of non-medical care was common. Among respondents, 29% reporting draining infections without seeking medical care, and 22.8% reported obtaining antibiotics through non-medical sources. Rates of self-management such as not seeking medical care and obtaining antibiotics outside of healthcare channels were nearly double in those who engaged in higher-risk injection practices.

## Discussion

These survey results offer an important national sample of the range of drug use and infection prevention practices currently employed by people who use drugs. For patients presenting with IDU-associated infections a careful history should include questions about drug preparation practices. These might include details of what solvents are used, skin hygiene practices prior to preparing drugs and/or injecting, how injection sites are prepared, use of saliva to lubricate needles, needle sharing practices, and an individual’s access to clean needles [24].

Two important question included in this survey which have not received any attention in the past were (1) if respondents had ever reused needles they previously used to drain an abscess to subsequently inject drugs or (2) if they had ever shared needles with someone else that had a fever or was sick. Alarmingly, these practices were common in our population. Clinicians should recognize that limited access to needles may result in individuals engaging in higher-risk practices and should consider asking PWID about these scenarios. This may be particularly relevant for PWID who present with recurrent infections which could be the result of repeated self-inoculation as might happen if needles used to lance abscesses are subsequently reused to inject drugs. This is particularly important for those with *S. aureus* infections as *S. aureus* is known to survive on fomites including injection drug use equipment [25] for up to two months [26] and is the most common cause of skin and soft tissue infections in this population [27]. Similarly, clinicians who elicit a history of PWID sharing needles with others who have fevers should view this as an opportunity to discuss the range of infections that can be spread through needle sharing, as well as use this as an opportunity to engage PWID in conversations about preventative healthcare including immunizations against hepatitis A and B, and pre-exposure prophylaxis for HIV.

We observed that respondents who reported having used harm reduction practices including cleaning injection sites with alcohol and cleaning used needles with bleach or alcohol were more likely to have experienced a bacterial or fungal injection site infection. Interestingly, this same group who reported using harm reduction techniques was also more likely to have ever engaged in all forms of non-sterile drug preparation practices. Because the survey did not assess timing of when respondents employed these practices relative to when they experienced injection site infections it is unclear if these harm reduction practices started prior to developing infections, or if they began engaging in safer practices as a result of having experienced complications in the past. One potential hypothesis is that those who experienced any type of injection-related infection may have received harm reduction education in conjunction with other medical care accounting for the higher use of these infection prevention practices in this group. Further research focused on the time frame during which specific injection practices were used, and the timing of injection-associated infections is needed.

Evidence-based harm reduction education on safer injection strategies should form a key component of preventative care for people who use drugs. Common-sense infection prevention principles such as washing hands, using clean needles, and educating patients about infectious diseases risks such as HIV and HCV need to be discussed when caring for patients with injection-associated infections [20, 28]. A recent national survey of infectious diseases physicians through the Emerging Infections Network highlighted that while ID physicians self-reported agreeing with harm reduction principles, many did not routinely incorporate counseling on safer injection strategies into the care of PWID who present with bacterial or fungal infections [29]. We suggest that physicians caring for people who inject drugs familiarize themselves with common injection drug use-related practices to provide infection prevention and harm reduction advice to their patients. Clinicians should work with hospitals to develop multidisciplinary teams based on local resources to ensure that PWID receive educational materials, adequate screening for infectious diseases, obtain access to medication treatment if interested, and undergo counseling to reduce their risk of future infections and hospitalizations. Given the high incidence of untreated mental health comorbidities among PWID, these interactions with the healthcare system also represent a key opportunity to link PWID to mental healthcare. Other allied health professionals, such as peer recovery specialists, nurse educators, and pharmacists, may also be able to provide counseling, education, and screening for infections. Multidisciplinary care teams have been successful in both inpatient and outpatient settings and provide a model for hospitals looking to improve the care of PWID [28, 30]. Furthermore, multidisciplinary teams may be able to provide individualized care plans that address common comorbidities among PWID, such as lack of access to safe housing, comorbid psychiatric conditions, and history of trauma [31].

Many respondents in this survey reported receiving medical care outside of a hospital or healthcare facility. While the limitations of this survey do not allow for more qualitative explorations on individual reasons why PWID may avoid healthcare institutions during acute illnesses, prior research in this area has identified that stigma [32], negative experiences of pain and withdrawal [33], and traumatic past experiences within the formal medical system [34], can all create barriers to infectious diseases care for PWID. Syringe service programs (SSPs), also referred to as needle exchanges or needle and syringe programs, have been established in several countries and may help bridge this care gap for PWID [35]. In the USA, there continues to be a complicated regulatory landscape posed by state and local drug paraphernalia laws that hinders expansion of SSPs into many states and limits adequate access to sterile injection supplies for many PWID [36]. Most SSPs offer free or low-cost harm reduction services such as naloxone rescue kits, education, infectious disease screening and vaccination, wound care, and recovery resources [37, 38]. SSPs may serve a critical role in not only providing access to clean injection supplies—directly addressing the high rates of sharing and reuse identified in this survey, but can also offer a low-barrier entry into healthcare including screenings for undiagnosed infectious diseases such as HIV and HCV that is often more acceptable to PWID [39, 40].

One noteworthy data point collected in this survey was high self-reported rates of sexually transmitted infections (STIs) including gonorrhea, chlamydia, and syphilis. PWID are at an increased risk for STIs [41, 42]. Prior research has suggested that in many regions of the USA there is an important epidemiologic link with between rates of syphilis [43] and substance use [44]. Recognizing this trend, the CDC has suggested that non-traditional healthcare settings, including acute hospitalization or other community health settings, provide PWID service bundles that include key aspects of targeted preventative healthcare including testing and treatment for infectious diseases including STIs, viral hepatitis, and HIV [45]. Clinicians should also offer immunizations for hepatitis A and B, and HIV pre-exposure prophylaxis [46]. This data on high rates of STIs, viral hepatitis, and HIV within a population entering treatment for opioid use disorder in 2021 underscores the importance of these recommendations. Screening for bacterial STIs and transactional sex in PWID entering substance use disorder treatment programs or hospitalized for other substance use-associated complications may represent an important opportunity to reduce the transmission of STIs within this population [47].

Our study has several limitations. First, SKIP is a sample of patients entering opioid use disorder treatment. While many patients report co-use of other substances, our results may be less generalizable to people who inject other drugs but do not inject opioids. Second, it is likely that our findings may be an underestimate of real-world injection site-related complications as this survey is limited by survivor bias—that is only participants who survive long enough to make it into opioid use disorder treatment participated in the study. Third, we relied on self-reports which are subject to recall bias. It is possible that some respondents may have limited understanding of the type of infections they have previously experienced and may have under-reported more severe infections such as bloodstream infections or endocarditis if they were less familiar with the names of these infections.

## Conclusion

Patients entering treatment for opioid use disorder commonly report non-sterile drug preparation practices, injection-associated infections, in a large national survey. Opioid use disorder treatment clinics may be important sites for harm reduction beyond overdose education. These may include educating patients about drug preparation practices, sexually transmitted infections, vaccination, and injection site infections.

## Data Availability

The datasets used and/or analyzed during the current study are available from the corresponding author on reasonable request.
